# CD8^+^NKT-like cells regulate the immune response by killing antigen-bearing DCs

**DOI:** 10.1038/srep14124

**Published:** 2015-09-15

**Authors:** Chao Wang, Xi Liu, Zhengyuan Li, Yijie Chai, Yunfeng Jiang, Qian Wang, Yewei Ji, Zhongli Zhu, Ying Wan, Zhenglong Yuan, Zhijie Chang, Minghui Zhang

**Affiliations:** 1 Institute of Immunology, Tsinghua University School of Medicine, Beijing 100084, China; 2 Institute of Immunology, Taishan Medical University, Taian, Shandong 271000, China; 3 Institute of Immunology, Third Military Medical University, Chongqing 400038, China

## Abstract

CD1d-dependent NKT cells have been extensively studied; however, the function of CD8^+^NKT-like cells, which are CD1d-independent T cells with NK markers, remains unknown. Here, we report that CD1d-independent CD8^+^NKT-like cells, which express both T cell markers (TCRβ and CD3) and NK cell receptors (NK1.1, CD49b and NKG2D), are activated and significantly expanded in mice immunized with GFP-expressing dendritic cells. Distinct from CD1d-dependent NKT cells, CD8^+^NKT-like cells possess a diverse repertoire of TCRs and secrete high levels of IFN-gamma but not IL-4. CD8^+^NKT-like cell development is normal in CD1d^−/−^ mice, which suggests that CD8^+^NKT-like cells undergo a unique development pathway that differs from iNKT cells. Further functional analyses show that CD8^+^NKT-like cells suppress T-cell responses through elimination of dendritic cells in an antigen-specific manner. Adoptive transfer of antigen-specific CD8^+^NKT-like cells into RIP-OVA mice prevented subsequent development of diabetes in the animals induced by activated OT-I CD8 T cells. Our study suggests that CD8^+^NKT-like cells can function as antigen-specific suppressive cells to regulate the immune response through killing antigen-bearing DCs. Antigen-specific down regulation may provide an active and precise method for constraining an excessive immune response and avoiding bypass suppression of necessary immune responses to other antigens.

Immune regulation plays an important role in maintaining immune homeostasis and provides necessary protection from tissue damage caused by excessive immune responses. Immunologists have documented many different types of immune regulation mechanisms that involve both cell types (e.g., Treg^1^, DCreg^2^, *et al.*) and various molecules (e.g., IL-10^3^, TGF-β^4^, NO^5^, Fas/FasL^6^, *et al.*)^7^; of such cells, NKT cells are particularly interesting due to their unique cell surface features and powerful role in the immune system. A special subset of immunocytes with both NK and T cell lineage markers, NKT cells have fascinated immunologists since their discovery in 1987^8^,^9^,^10^. NKT cells can be divided into two major subtypes based on their CD1d dependence^11^: CD1d-dependent NKT cells with biased TCR recognizing lipid antigens (e.g., α-GalCer) and CD1d-independent NKT cells with diverse TCRs. CD1d-dependent NKT cells, especially invariant NKT (iNKT) cells, have been intensively investigated in recent years after CD1d^−/−^ mice^12^ and the CD1d tetramer were created^13^. Studies suggest that iNKT cells are involved in multiple aspects of the immune response, including inflammation, tumor immunology and immune regulation^14^. iNKTs use two different methods for their regulatory activities: activated iNKTs secrete high levels of IFN-γ or IL-4 to promote Th1 or Th2 responses^15^, and they directly interact with DCs^14^.

An examination of NKT cell subsets in CD1d^−/−^ mice revealed the CD1d-independent NKT cell subset; approximately 50% of the cells therein express CD8^16^. This CD8-expressing CD1d-independent NKT cell subset has been referred to as “CD8^+^NKT cells” in some previous studies^17^,^18^,^19^. However, the name “CD8^+^NKT cells” is intriguing to immunologists interested in CD1d-dependent NKT cells. To avoid controversy and enhance accuracy, we use “CD8^+^NKT-like cells” herein instead of “CD8^+^NKT cells” to indicate CD8-expressing, CD1d-independent NKT cells, which was suggested by Godfrey^12^ for NKT cell subset nomenclature. Previous studies on the CD8^+^NKT-like cell subset have mainly focused on antitumor effects and demonstrate that these cells from both MHC-I-competent^18^,^20^ and -deficient mice^19^ produce high levels of IFN-γ and exhibit an effective tumoricidal capacity against tumor cells. However, an immunoregulatory function has not been described, and the immunological features must also be comprehensively characterized.

In this study, we focused on the CD1d-independent CD8^+^NKT-like cell subset and found that the cell number increased after vaccination with LPS-pulsed, GFP-expressing dendritic cells (GFP-DCs, wherein GFP can be presented as endogenous antigen). Combined with evidence of their phenotypic, developmental and functional features, we conclude that CD8^+^NKT-like cells are distinct from previously identified iNKT and classical CD8 T cells; furthermore, the cells suppress the immune response in an antigen-specific manner by effectively killing antigen-bearing DCs.

## Results

### The CD8^+^NKT-like cell number increased after immunization

**Figure 1:**
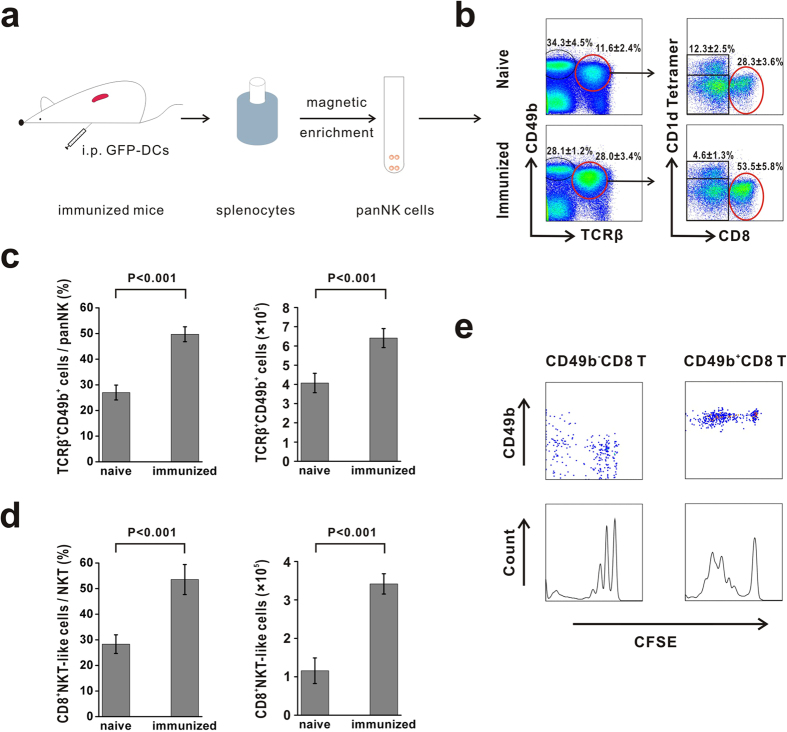
The number of CD8^+^NKT-like cells increased after immunization with GFP-DCs. (**a**) The flow chart shows that C57BL/6 mice were immunized by 2 × 10^6^ LPS-pulsed GFP-DCs four times. The splenocytes were then isolated, and panNK cells were enriched using a panNK selection kit. (**b**) The gating strategy used to identify NK and NKT cell subsets is shown. (**c**) The proportion of TCRβ^+^CD49b^+^ cells in panNK cells and the absolute number of TCRβ^+^CD49b^+^ cells in naïve mice and GFP-DC-immunized mice were compared. (**d**) The proportion of CD8^+^NKT-like cells in TCRβ^+^CD49b^+^ cells and the absolute number of CD8^+^NKT-like cells in naïve mice and GFP-DC-immunized mice were compared. (**e**) We isolated 1 × 10^6^ CD49b^+^CD8 T cells and 1 × 10^7^ CD49b^−^CD8 T cells from B6 CD45.1 mice and B6 Thy1.1 mice, respectively, through flow cytometry; the cells were injected into C57BL/6 mice, which were subsequently immunized with 2 × 10^6^ GFP-DCs. After 14 days, the recipient mice were again immunized with 2 × 10^6^ GFP-DCs. Next, donor splenic cells were analyzed for phenotype, and the CFSE dilution experiment was performed after 3 days. The data represent three independent experiments (n = 8–10).

To determine changes in the C57BL/6 mouse NKT cell subsets after immunization with LPS-pulsed GFP-DCs, panNK (CD49b^+^) cells were magnetically enriched and used in the subsequent analyses (Fig. 1a). NKT cells can be divided into three subsets based on CD8 expression and affinity to an α-GalCer-loaded CD1d tetramer: Tetramer^−^CD8^−^ NKT cells, Tetramer^−^CD8^+^NKT (CD8^+^NKT-like) cells and Tetramer^+^NKT (iNKT) cells (Fig. 1b, Supplementary Figure S1). In immunized mice, we found that the proportion of NKT cells in isolated panNK cells increased from 27.1 ± 2.9% to 49.7 ± 2.9%, while the number of NKT cells increased from 4.1 × 10^5^  ± 5.1 × 10^4^ to 6.4 × 10^5^  ± 4.9 × 10^4^ (Fig. 1c). Further analyses showed that the proportion of CD8^+^NKT-like cells in NKT cells increased from 28.3 ± 3.6% to 53.5 ± 5.8%, while the number of CD8^+^NKT-like cells increased from 1.2 × 10^5^ ± 3.3 × 10^4^ to 3.4 × 10^5^ ± 2.7 × 10^4^ after immunization (Fig. 1d). Considering the close relationship between CD8-expressing lymphocytes and DCs loaded with an endogenous antigen, we speculated that CD8^+^NKT-like cells are activated and the cell number is increased by GFP-DCs.

The increased number of CD8^+^NKT-like cells may be due to either CD8^+^NKT-like cell proliferation or acquisition of NK cell markers by conventional CD8 T cells. To examine the two possibilities in our model, we performed adoptive transfer experiments using CD45.1^+^CD49b^+^CD8 T cells or CD90.1^+^CD49b^−^CD8 T cells to clarify the origin of the increased CD49B^+^CD8 T cell number. Wild type CD45.2^+^ mice were injected with CFSE-labeled CD45.1^+^CD49b^+^CD8 T cells (sorted from B6 CD45.1 mice, purity >95%) or CD90.1^+^CD49b^−^CD8 T cells (sorted from B6 Thy1.1 mice, purity >98%), which was followed by two injections of GFP-DCs. Next, the recipient mice were sacrificed, and splenic panNK cells were magnetically sorted and analyzed through flow cytometry. The data show that the donor-derived CD45.1^+^CD49b^+^CD8 T cells proliferated after a GFP-DCs challenge and retained the CD49b^+^CD8^+^ phenotype. However, the donor-derived CD90.1^+^CD49b^−^CD8 T cells proliferated, and a population acquired a CD49b marker after GFP-DC immunization (Fig. 1e), which suggests that the increased number of CD8^+^NKT-like cells were generated from 1) CD8^+^NKT-like cell proliferation and 2) acquisition of a CD49b marker by conventional CD8 T cells.

### CD8^+^NKT-like cell phenotype

**Figure 2:**
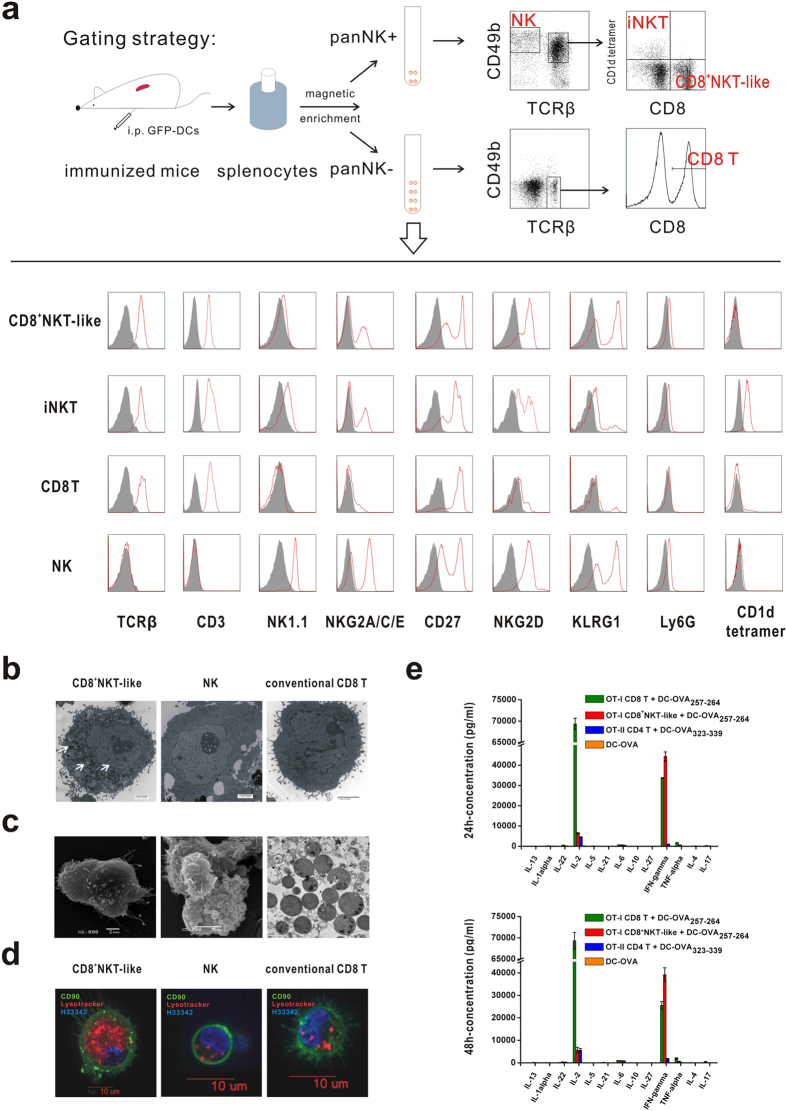
CD8^+^NKT-like cell phenotype. (**a**) CD8^+^NKT-like cell phenotypes were compared with CD8 T cells, NK cells and invariant NKT cells; the red line indicates the expression level, and the gray-filled histogram shows the corresponding isotype. (**b**) The CD8^+^NKT-like cell, NK cell and conventional CD8 T cell morphologies were compared using a transmission electron microscope. (**c**) Intact (left) and mechanically fragmented (middle) CD8^+^NKT-like cells were detected using a scanning electron microscope. Next, granules from mechanically fragmented CD8^+^NKT-like cells were visualized using a transmission electron microscope (right). (**d**) CD8^+^NKT-like cells, NK cells and conventional CD8 T cells were stained with CD90.2-FITC (green), LysoTracker Red (red) and Hoechst 33342 (blue). Images were collected through Andor live cell confocal microscopy; the scale bars are shown. (**e**) CD4 T cells were separated and sorted from OT-II mice, while CD8 T cells and CD8^+^NKT-like cells were isolated and then sorted from OT-I mice. The cells were co-cultured with DCs loaded with corresponding peptides, respectively, and the supernatant was collected and detected using a CBA assay at the indicated time points. These data are representative of four independent experiments (n = 8).

To explore the characteristics of the CD8^+^NKT-like cells, the CD8^+^NKT-like cell phenotype was compared with CD8 T cell, NK cell and invariant NKT cell phenotypes (Fig. 2a). The data show that the CD8^+^NKT-like cell subset co-expressed T cell markers (e.g., CD3 and TCRβ) and NK cell markers (e.g., NK1.1, CD49b, NKG2D, NKG2A/C/E, KLRG1, *et al.*). Compared with iNKT cells, CD8^+^NKT-like cells could not bind to an α-GalCer-loaded CD1d tetramer. In addition, the conventional CD8 T cells did not express NK cell markers, which were expressed on the CD8^+^NKT-like cells and NK cells. These data demonstrate that CD8^+^NKT-like cells differ phenotypically from iNKT cells and conventional CD8 T cells. We also tested whether the CD8^+^NKT-like cells expanded *in vitro* through co-culturing sorted splenic panNK cells with GFP-DCs in the presence of IL-2, IL-7 and IL-15. The cells that emerged from the co-culture system exhibited a phenotype similar to the cells generated *in vivo* (Supplementary Figure S2). To characterize the CD8^+^NKT-like cells, we compared the CD8^+^NKT-like cell, NK cell and conventional CD8 T cell morphologies using TEM, which provided visual evidence that the CD8^+^NKT-like cells were larger than the NK cells as well as conventional CD8 T cells and that the CD8^+^NKT-like cells contained more granules (white arrows, Fig. 2b). EM images of intact and fragmented CD8^+^NKT-like cells revealed abundant granules that were 1 μm in diameter (Fig. 2c). Confocal microscopy images also showed that CD8^+^NKT-like cells exhibited lower nucleus-cytoplasmic ratios, and the cytoplasm contained more granules, which was indicated by the lysosome-staining dye LysoTracker (Fig. 2d), suggesting a potential cytotoxic capacity. To further explore the cytokine profile, CD4 T cells from OT-II mice and CD8 T cells and CD8^+^NKT-like cells from OT-I mice were sorted (purity > 95%, see Supplementary Figure S3) and co-cultured with DCs loaded with the corresponding peptides, respectively; the supernatants were collected and examined at the indicated time points. Unlike iNKT cells, which regulate the immune response by secreting an abundance of cytokines (e.g., IFN-γ and IL-4), the CD8^+^NKT-like cells secreted the highest levels of IFN-γ when stimulated by TCR-matched antigens (Fig. 2e). The limited CD8^+^NKT-like cell cytokine profiles demonstrated a functional distinction compared with iNKT cells.

### CD8^+^NKT-like cell TCR categories

**Figure 3:**
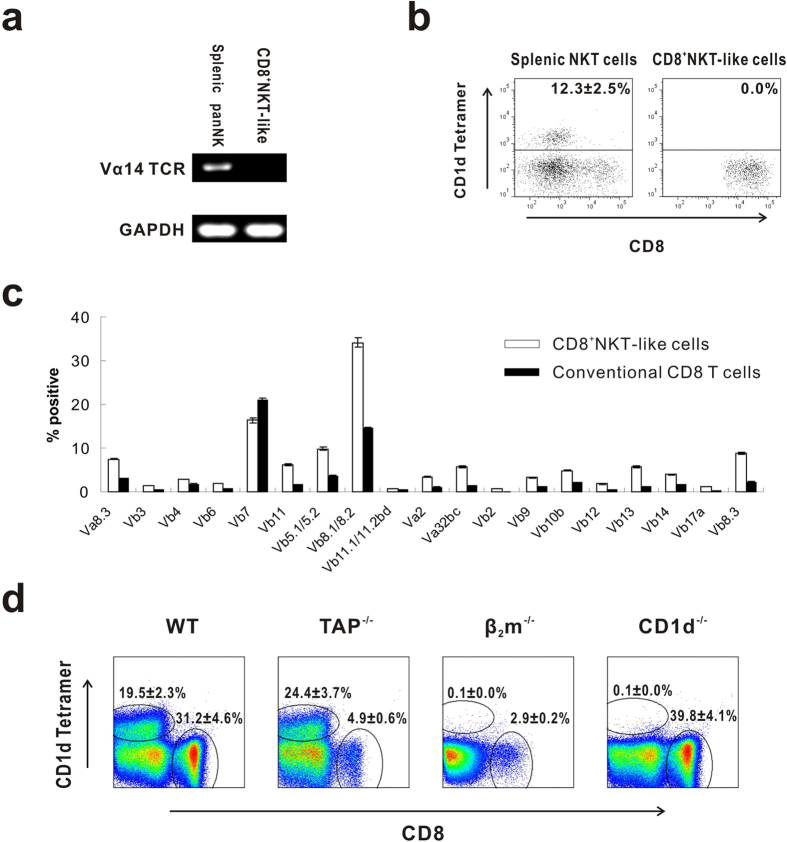
The CD8^+^NKT-like cells are distinct from the iNKT cells. (**a**) The Va14 expression level was detected in CD8^+^NKT-like cells, and splenic panNK cells were used as the positive control. (**b**) Freshly isolated splenic TCRβ^+^CD49b^+^ cells (NKT cells) and CD8^+^NKT-like cells were stained with α-GalCer-loaded CD1d tetramer and analyzed using a FACSAria II. (**c**) The different types of TCRs in CD8^+^NKT-like cells and conventional CD8 T cells were compared. (**d**) The CD8^+^NKT-like cell populations from magnetically isolated panNK cells in C57BL/6, CD1d^−/−^, β_2_m^−/−^ and TAP^−/−^ mice are shown. These data are representative of three independent experiments (n = 8).

iNKT cells are defined by biased Vα14 TCR expression and an affinity for the α-GalCer-loaded CD1d tetramer. To distinguish CD8^+^NKT-like cells from iNKT cells, we used the Vα14 TCR with a PCR assay to demonstrate that CD8^+^NKT-like cells do not express the invariant Vα14 TCR chain (Fig. 3a). CD8^+^NKT-like cells were also negative upon α-GalCer-loaded CD1d tetramer staining (Fig. 3b). Next, we characterized the CD8^+^NKT-like cell TCR profiles and found that CD8^+^NKT-like cells possess a diverse TCR repertoire, which is comparable to conventional CD8 T cells (Fig. 3c). The CD8^+^NKT-like cell TCR diversity suggests that the cells recognize different antigen epitopes, including but not limited to lipid antigen presented by other cells, such as dendritic cells. The interaction between these cells may provide physiological and pathological functions.

To characterize the developmental features of CD8^+^NKT-like cells, we examined their presence in CD1d^−/−^ mice, TAP^−/−^ mice, and β_2_ m^−/−^ mice. CD1d^−/−^ mice do not contain the MHC-I-related molecule CD1d, which is necessary for iNKT cell development. TAP^−/−^ mice exhibit severely reduced levels of surface MHC-I molecules, while β_2_m^−/−^ mice lack surface expression of both CD1d and the MHC-I molecule. Our data show that the CD8^+^NKT-like cells are normal in CD1d-deficient mice but are significantly impaired in both TAP^−/−^ mice and β_2_m^−/−^ mice (Fig. 3d), which suggests that CD8^+^NKT-like cell development is independent of CD1d, but it requires the MHC-I molecule.

### CD8^+^NKT-like cell functional features

**Figure 4:**
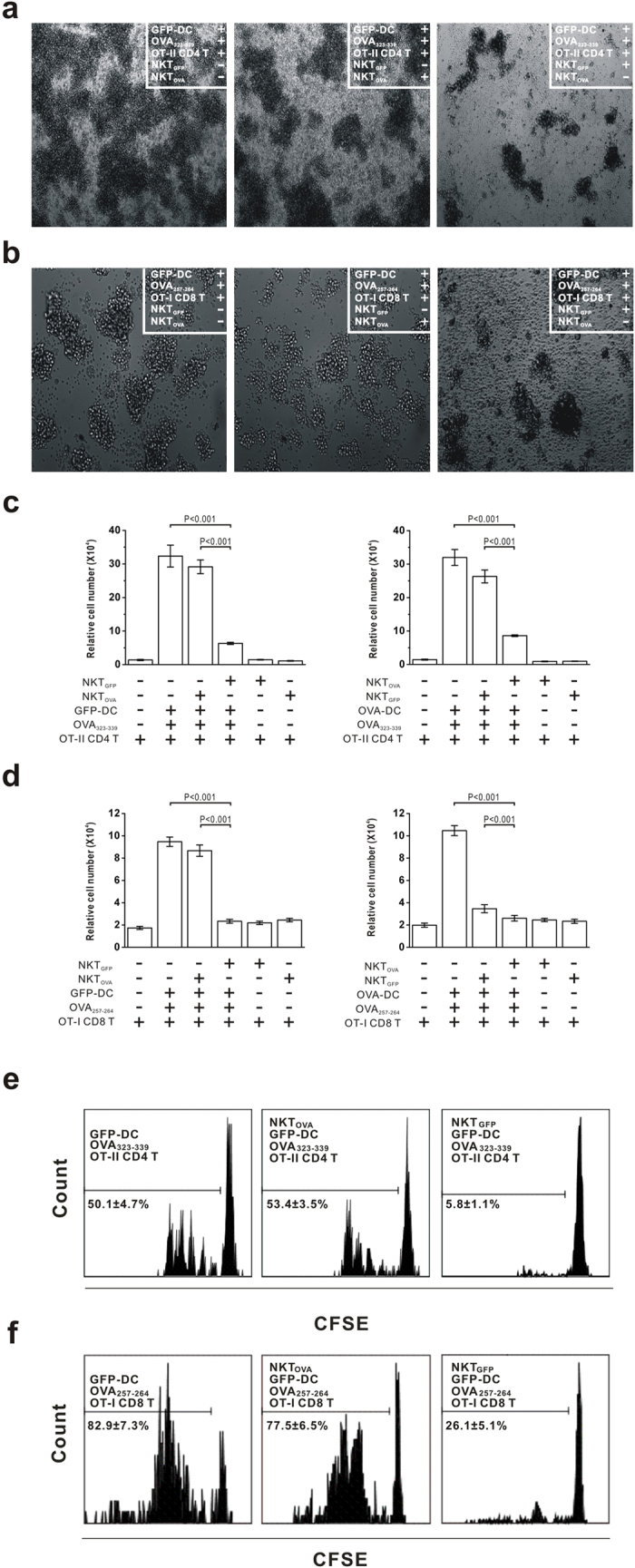
CD8^+^NKT-like cells inhibited T cell responses in an antigen-specific manner. (**a**) NKT_GFP_ suppressed proliferation of OT-II-specific CD4 T cells activated by GFP-DCs loaded with OT-II peptides *in vitro*. (**b**) NKT_GFP_ suppressed the proliferation of OT-I-specific CD8 T cells activated by GFP-DCs loaded with OT-I peptides *in vitro*. Photographs were taken with a digital imaging system (objective, 10×). (**c**) NKT_GFP_ and NKT_OVA_ inhibited OT-II-specific CD4 T cell responses activated by OT-II-loaded GFP-DCs and OVA-DCs, respectively. (**d**) NKT_GFP_ and NKT_OVA_ inhibited OT-I-specific CD8 T cell responses activated by OT-I-loaded GFP-DCs and OVA-DCs, respectively. (**e**) NKT_GFP_ or NKT_OVA_ were adoptively transferred, and the CFSE dilution was detected on GFP-DC-presenting CD4 T cells. f. NKT_GFP_ or NKT_OVA_ were adoptively transferred, and the CFSE dilution was detected on GFP-DC-presenting CD8 T cells. NKT_GFP_, CD8^+^NKT-like cells induced from GFP-DC immunized mice. NKT_OVA_, CD8^+^NKT-like cells from OVA-DC immunized mice. The data are representative of three independent experiments.

The iNKT cell immune-regulatory roles prompted us to explore the potential regulatory functions of CD8^+^NKT-like cells. CD8^+^NKT-like cells were added to an *in vitro* immune response system, wherein OVA-specific TCR transgenic T cells were used as responders, and GFP-DCs or OVA-DCs loaded with corresponding peptides were used as antigen-presenting stimulators. We found that CD8^+^NKT-like cells from GFP-DC-immunized mice (NKT_GFP_), but not OVA-DC-immunized mice (NKT_OVA_), aborted antigen-specific CD4 T (Fig. 4a) and CD8 T (Fig. 4b) cell proliferation if GFP-DCs were used as antigen-presenting cells, and vice versa. Corresponding analyses on the number of OT-II-specific CD4 T cells (Fig. 4c) and OT-I-specific CD8 T cells (Fig. 4d) are also shown. These data suggest that adding CD8^+^NKT-like cells inhibited DC-mediated CD4 and CD8 T-cell responses in an antigen-specific manner.

To confirm the antigen-specific CD8^+^NKT-like cell inhibitory functions *in vivo*, OVA-specific TCR transgenic CD4 and CD8 T cells were intraperitoneally pre-injected into C57BL/6 mice with NKT_GFP_ or NKT_OVA_ cells. Next, the mice were challenged by GFP-DCs loaded with OT-II or OT-I peptides. In the mice pretreated with NKT_GFP_, DC immunization did not stimulate CD4 T cell (Fig. 4e) or CD8 T cell (Fig. 4f) proliferation, but T-cell proliferation was normal in mice pretreated with NKT_OVA_. These data indicate that the CD8^+^NKT-like cell-mediated T-cell response suppression requires dendritic cell presentation of the proper endogenous antigen, which suggests antigen-specific recognition and subsequent regulation of DCs by CD8^+^NKT-like cells.

### Cytotoxicity against DCs by CD8^+^NKT-like cells

**Figure 5:**
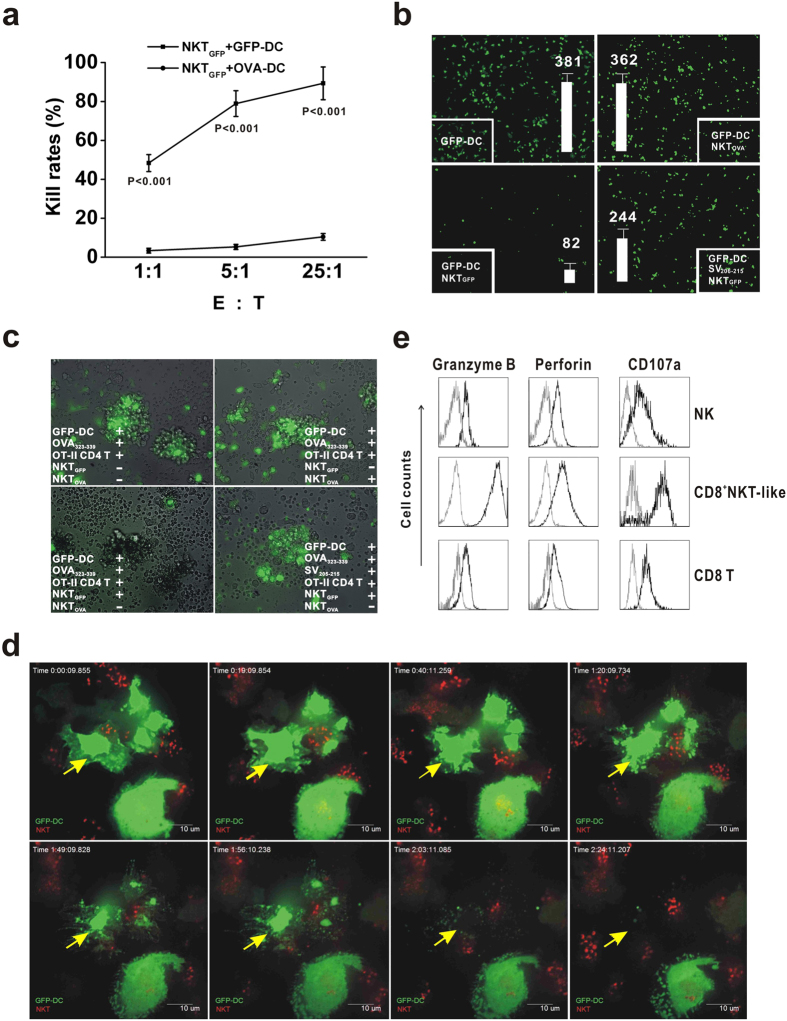
CD8^+^NKT-like cells inhibited the T-cell response by killing DCs. (**a**) NKT_GFP_ cells were co-cultured with GFP-DCs or OVA-DCs (stained with CMFDA) at the indicated E:T ratios. (**b**) GFP-DCs (green) were co-cultured with NKT_GFP_ or NKT_OVA_ cells at the E:T ratio 5:1 for 12 hours and observed using a Deltavision deconvolution microscope. The right bottom panel shows that adding 2 μg/ml SV_206-215_ peptides affects NKT_GFP_-mediated GFP-DC elimination. The number of GFP-DCs was calculated and is shown as white bars. (**c**) The presentation of OT-II peptides to OT-II-specific CD4 T cells by GFP-DCs with intervention by CD8^+^NKT-like cells is highlighted. (**d**) Dynamic display of the NKT_GFP_-mediated GFP-DC (green) killing process; NKT_GFP_ cells are labeled with LysoTracker Red (red), and the yellow arrows indicate morphologic changes in GFP-DCs. The images were collected using Andor live cell confocal microscopy using a 60× oil immersion lens. (**e**) The expression levels for granzyme B, perforin and CD107a were examined among the CD8^+^NKT-like cells, NK cells and CD8 T cells using intracellular cell staining assays. The results are representative of three independent experiments.

We hypothesized that the interaction between CD8^+^NKT-like cells and antigen-bearing DCs is responsible for the immunosuppressive effect. To examine this possibility, these two types of immunocytes were co-cultured *in vitro*. We observed that the number of GFP-DCs, not OVA-DCs, significantly decreased after 12 hours of co-culture with NKT_GFP_ (Fig. 5a), which indicates rapid antigen-specific cytotoxicity of NKT_GFP_ toward GFP-DCs. To further clarify how CD8^+^NKT-like cells kill DCs, GFP-DCs were co-cultured with NKT_GFP_ or NKT_OVA_ *in vitro*, and the interaction between these cells was investigated using a Deltavision Deconvolution Microscope. We observed direct and rapid killing of GFP-DCs by NKT_GFP_, but not NKT_OVA_ (Fig. 5b). These data suggest that CD8^+^NKT-like cells require antigen-specific recognition to kill DCs. To clarify, a high concentration (2 μg/ml) of unrelated SV_205–215_ peptides^21^, which competitively bind to MHC-I molecules on GFP-DCs, was added to the co-culture system, and the results show that the peptide effectively inhibited NKT_GFP_-mediated cytotoxicity toward GFP-DCs (Fig. 5b). Accordingly, SV_205–215_ also prevented GFP-DC killing by NKT_GFP_ and restored OVA-specific CD4 T-cell proliferation (Fig. 5c). These data suggest that CD8^+^NKT-like cells recognize MHC-I-binding peptides and exert a cytotoxic effect on DCs in an MHC-I-restricted manner (Supplementary Figure S4). Live cell imaging shows that LysoTracker-labeled NKT_GFP_ cells (red) can recognize and kill GFP-DCs (green) within 2 hours (Fig. 5d, Supplementary Video S1), which is an effective and rapid approach to eliminating antigen-bearing DCs by CD8^+^NKT-like cells, which might help terminate an immune response.

To illustrate how CD8^+^NKT-like cells kill antigen-bearing DCs, we detected the expression levels of effector molecules involved in the CD8^+^NKT-like cell death pathway. The data revealed that the cells express higher levels of granzyme B, perforin and CD107a (Lamp-1) compared with NK cells or conventional CD8 T cells (Fig. 5e), which indicates that the granule exocytosis pathway is responsible for CD8^+^NKT-like cell-mediated cytotoxicity against DCs. GeneChip data also demonstrate that CD8^+^NKT-like cells express more granule exocytosis-associated molecules than NK or conventional CD8 T cells (Supplementary Figure S5). These data suggest that the granule exocytosis pathway mediates antigen-bearing DC elimination by CD8^+^NKT-like cells.

### The physiological roles of CD8^+^NKT-like cells

**Figure 6:**
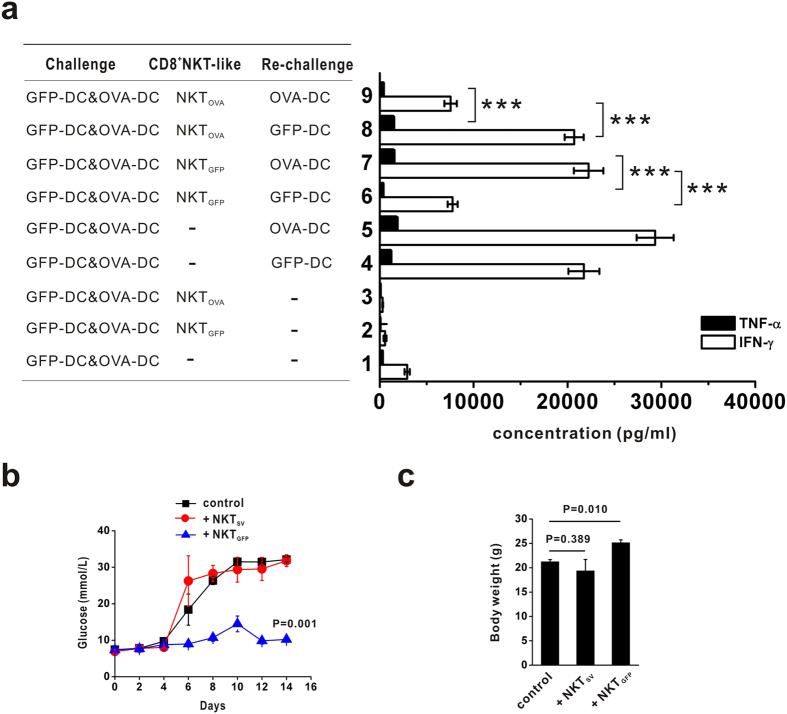
CD8^+^NKT-like cells involved in immune downregulation *in vivo*. (**a**) NKT_GFP_ or NKT_OVA_ cells were injected into naïve mice, which subsequently received immunization with GFP-DCs and OVA-DCs. Two weeks later, splenocytes from each group were co-cultured with GFP-DCs or OVA-DCs, and the TNF-alpha and IFN-gamma production levels were detected in the supernatant at 72 hours. These data are representative of three independent experiments (n = 8). (**b**,**c**) NKT_GFP_ and NKT_SV_ cells were pre-injected into RIP-OVA mice, which was subsequently injected peritoneally with OVA-specific CD8 T cells and GFP-DCs loaded with OT-I peptides. The blood glucose dynamics (**b**) and body weight (**c**) were measured in both conditions. These data are representative of two independent experiments (n = 8). ***P < 0.001.

Originally, we showed that GFP-DC immunization increased the number of CD8^+^ NKT_GFP_ cells in mice. The *in vitro* data also suggested that CD8^+^ NKT-like cells might play a role in regulating DCs. Therefore, we further explored the regulatory function of CD8^+^ NKT-like cells in the immune response *in vivo*. The mice were first injected with NKT_GFP_ or NKT_OVA_ cells and subsequently received an immunization with both GFP-DCs and OVA-DCs. Two weeks later, splenocytes from each group were co-cultured with GFP-DCs or OVA-DCs for 72 h, and the level of cytokines secreted in the supernatant was determined. We found that, if the mice were pre-treated with NKT_GFP_, the splenocyte re-challenge by GFP-DCs yielded lower levels of TNF-α and IFN-γ compared with the cells re-challenged by OVA-DCs. Similar results were observed in mice immunized with NKT_OVA_ cells (Fig. 6a). These data demonstrate that intervention with antigen-specific CD8^+^NKT-like cells kills specific antigen-bearing DCs and reduces their opportunity to stimulate immunocytes and down-regulate the immune response. RIP-OVA transgenic mice were also used to study the immunosuppressive effect of CD8^+^NKT-like cells. RIP-OVA transgenic mice, which express high levels of ovalbumin protein in their pancreas, were subjected to severe pancreatic islet damage when injected with CTLs activated by OVA_257–264_-loaded GFP-DCs and exhibited elevated blood glucose levels as well as lower body weight. However, pre-injection with NKT_GFP_, but not NKT_SV_, significantly inhibited the blood glucose increase (Fig. 6b) and body weight decrease (Fig. 6c).

## Discussion

CD1d-dependent iNKT cells have attracted immunologists’ attention for decades because they are important for immune regulation. Although iNKT cells contain a biased TCR repertoire, which includes the invariant Vα14Jα18 chain, they are closely involved in immune regulation and affect multiple aspects of the immune response^11^. When stimulated with DCs that present the lipid antigen α-GalCer, iNKT cells produce high levels of cytokines, including IL-4, IFN-γ and TNF-α, which promote Th1 or Th2 responses^14^. The interaction between iNKT cells and DCs may also upregulate the levels of MHC molecules and co-stimulatory molecules (e.g., CD40, CD80, CD86, etc.) expressed on DCs, which then enhances T cell and iNKT cell activation^15^,^22^. Impaired virus clearance and tumor inhibition was demonstrated in Jα18-knockout mice, which are deficient in iNKT cells^23^,^24^, suggesting that iNKT cells play an important role in the immune response.

Our data suggest that, unlike iNKT cells, CD8^+^NKT-like cells include a diverse TCR repertoire and cannot bind to an α-GalCer-loaded CD1d tetramer. The high TCR diversity in CD8^+^NKT-like cells indicates the cells’ potential to recognize a variety of protein antigens presented by interacting cells. However, whether antigen-specific recognition is required for the cells to exert its effect was only preliminarily examined in this study, and our data suggest that CD8^+^NKT_GFP_ cells only recognize GFP-expressing DCs, not OVA-DCs. CD8^+^NKT-like cells also exhibit a unique cytokine profile with high levels of IFN-γ secretion, which is distinct from iNKT cells^15^. The precise role of the CD8^+^NKT-like cell-secreted IFN-γ was not studied in this work. Typically, IFN-γ favors Th1 responses and suppresses Th2 differentiation, which provides cellular immunity against pathogen-loaded cells. Unlike iNKT cells, which may promote either Th1 or Th2 responses under different circumstances, CD8^+^NKT-like cells might only favor the Th1 response. GFP-expressing DCs, which mimic intracellular antigen-loaded DCs, should induce a Th1 immune response. Thus, GFP-expressing DCs-activated CD8^+^NKT-like cells may also favor a Th1 response. We also show that the CD8^+^NKT-like cells exhibit abundant granules in their cytoplasm and express high levels of granzyme, perforin and LAMP-1, which suggests strong, direct CTL-like cytotoxicity activity in these cells. Our data show that CD8^+^NKT_GFP_ cells directly kill GFP-expressing DCs.

Previous studies have demonstrated that immunocytes with cytotoxic capacities can kill DCs. A subset of L-selectin^−^CCR7^−^ CD8 T cells can enter reactive lymph nodes and kill antigen-bearing DCs to terminate an immune response^25^. Studies have also shown that a subset of CD94/NKG2A^+^KIR^−^ NK cells can kill DCs to control a downstream adaptive immune response^26^. Here, we show that another cell type, CD8^+^NKT-like cells, can also kill dendritic cells to downregulate the immune response. Furthermore, we found that antigen-dependent recognition between CD8^+^NKT-like cells and DCs was required before the dendritic cells were killed. In both the *in vitro* and *in vivo* models, CD8^+^NKT_GFP_ cells downregulated antigen-specific T-cell responses through eliminating GFP-expressing DCs, but CD8^+^NKT_OVA_ did not exhibit such downregulation.

Our data show that CD8^+^NKT-like cells vigorously proliferate upon stimulation by antigen-load DCs (Supplementary Figure S6). The data also show that the increase in CD8^+^NKT-like cells lags behind conventional CD8 T cells, which may facilitate antigen-specific CD8^+^NKT-like cell proliferation first and then migration of the cells to inflammatory sites to kill antigen-matched DCs. The immune regulatory subset may also kill antigen-loaded DCs at the later immune response stage, which prevents an excessive immune response. Based on these observations, we propose a hypothesis to explain the physiological relevance of CD8^+^NKT-like cells (Supplementary Figure S7). Antigen-pulsed DCs not only initiate antigen-specific CD4 and CD8 T cells as the main effector cells, but they also activate antigen-specific CD8^+^NKT-like cells. The increase in antigen-specific CD8^+^NKT-like cells in the later immune response stage may control the immune reaction by killing antigen-bearing DCs, preventing an excessive immune response, which leads to autoimmune disease.

Immunologists have reported that a proportion of NK1.1^−^CD8 T cells may acquire NK1.1 and other NK-associated markers upon cytokine activation^27^. CD8-expressing NKT-like cells have also exhibited proliferative capability in the presence of IL-2 *in vitro*^19^. To discern the origin of the CD8^+^NKT-like cells in our model, we performed adoptive transfer experiments using CD45.1^+^CD49b^+^CD8 T cells or CD90.1^+^CD49b^−^CD8 T cells to determine the origin of the increased number of CD49b^+^CD8 T cells. The data suggest that the CD8^+^NKT-like cell number increase is due to both CD8^+^NKT-like cell proliferation and NK cell marker acquisition by conventional CD8 T cells. Our data also showed that CD8^+^NKT-like cell development is independent of CD1d, but the MHC-I molecule is required. Therefore, the developmental features of the special CD8 T cell subset that acquired NK cell markers must be explored further.

In our laboratory, we have also investigated the presence of human CD8^+^NKT-like cells, which are phenotypically consistent with CD8^+^NKT-like cells. The CD3^+^CD56^+^CD8^+^Vα24^−^ cell population was observed in human peripheral blood. The expression of CD3, CD56 and CD8 as well as the absence of the invariant NKT marker Vα24 are consistent with the mouse CD8^+^NKT-like cells that we discuss herein (unpublished data). The number of CD3^+^CD56^+^CD8^+^Vα24^−^ cells also decreased in both rheumatoid arthritis and SLE patients (unpublished data), which indicates a role in immune regulation.

Immune responses are initiated through antigen acquisition, processing, and presentation by dendritic cells. Next, antigen-specific T cells are stimulated, and effective immune responses are established for antigen clearance. Therefore, a delicate braking system should also be employed to eliminate DCs or to render DCs in a state of anergy to limit excessive immune responses. Researchers have used the following negative feedback approaches to target antigen-bearing DCs: (1) activation-induced cell death, (2) mature DC conversion into regulatory DCs and (3) lymphocyte-mediated direct cytotoxicity against DCs. Clearly, of the three approaches, antigen-bearing DC elimination is considered the most positive and effective way to arrest unwanted immune responses. In previous studies on immunoregulation and NKT cell subsets, we observed a population of CD1d-independent CD8-expressing NKT-like cells that inhibited CD4 and CD8 T-cell responses by recognizing and killing antigen-specific DCs. Therefore, as a special subset of NKT cells, CD8^+^NKT-like cells exert immunosuppressive effects on the immune response.

## Methods

### Media and reagents

RMPI 1640 medium (Gibco, MA) supplemented with 10% FCS (Gibco, MA) was used for *in vitro* lymphocyte culture. Recombinant mouse GM-CSF, IL-2, IL-4, IL-7 and IL-15 were obtained from PeproTech. The fluorescent dyes used for cell staining were CFSE, CMFDA, LysoTracker, and 7-AAD (Life Technologies, MA). The MHC-I-binding peptide SIINFEKL (OVA amino acids 257–264), SAINNYAQKL (SV amino acids 206–215) and MHC-II-binding peptide ISQAVHAAHAEINEAGR (OVA amino acids 323–339) were synthesized by the Chinese Peptide Company. LPS and other common biochemical reagents were purchased from Sigma.

### Mice

Both the wild-type and transgenic mouse strains, including B6.GFP mice [C57BL/6-Tg(ACTB-EGFP)1Osb/J mice], B6.OVA mice [C57BL/6-Tg(ACTB-OVA)916Jen/J mice], B6 Thy1.1 mice (B6.PL-Thy1^a^/CyJ mice), OT-I mice [C57BL/6-Tg(TcraTcrb)1100Mjb/J mice], OT-II mice [B6.Cg-Tg(TcraTcrb)425Cbn/J mice], CD1d^−/−^ mice [B6.129S6-Del(3Cd1d2-Cd1d1)1Sbp/J mice], β_2_m^−/−^ mice (B6.129P2-B2m^tm1Unc^/J mice) and TAP^−/−^ mice (B6.129S2-Tap1^tm1Arp^/J mice) were obtained from the Jackson Laboratory and bred in specific pathogen-free conditions. The mice were used at 6–10 weeks old. The mice were treated in accordance with the National Institute of Health Guide for the Care and Use of Laboratory Animals with the approval of the Scientific Investigation Board of Tsinghua University, Beijing.

### Preparation of mature DCs

Mature DCs from bone marrow were cultured with recombinant mouse GM-CSF and IL-4 using established methods^2^ and were stimulated with 1 ng/ml LPS before use.

### Flow cytometric analysis

To analyze the lymphocyte subsets and assess surface marker expression, the lymphocytes were stained with a FITC-conjugated anti-TCRβ antibody, a PerCP-conjugated anti-NK1.1 antibody, a PE-conjugated anti-CD49b antibody, an APC-conjugated anti-CD4 antibody, an APC-Cy7-conjugated anti-CD8 antibody, a PE-conjugated anti-CD25 antibody, a FITC-conjugated anti-CD44 antibody, a PE-conjugated anti-CD62 L antibody, a FITC-conjugated anti-CD69 antibody, a PE-conjugated anti-CD107a antibody, an APC-conjugated anti-KLRG1 antibody, a PE-Cy7-conjugated anti-NKG2D antibody (eBioscience, CA), a FITC-conjugated anti-Ly6G antibody, a FITC-conjugated anti-CD27 antibody, a Brilliant Violet 510-conjugated anti-CD4 antibody, a Brilliant Violet 605-conjugated anti-CD8 antibody (Biolegend, CA), a FITC-conjugated anti-NKG2A/C/E antibody and fluorescent antibodies to various types of TCRs (BD Pharmingen, CA). Absolute cell counts were performed using counting beads (Spherotech, IL). Flow cytometry was performed using a FACSAria II (Becton Dickinson, CA), and the data were analyzed using FlowJo software.

### *In vitro* CD8^+^NKT-like cell amplification

After intraperitoneal immunization with 2 × 10^6^ GFP-DCs 4 times, the mice were sacrificed, and the splenocytes were harvested on day 7. The CD49b^+^panNK cells were isolated using the panNK Selection Kit (Stemcell, BC, Canada). Next, the positive cells were co-cultured in RMPI 1640 medium supplemented with 10% FCS, 50 ng/ml recombinant mouse IL-2, 10 ng/ml recombinant mouse IL-7, 50 ng/ml recombinant mouse IL-15 and GFP-DCs (at 5 × 10^4^ per 10^6^ panNK cells) for 14 days. The amplified lymphocytes were collected, and the CD8^+^CD49b^+^TCRβ^+^ cells (CD8^+^NKT-like cells) were sorted using FACSAria II flow cytometry. The phenotype of the CD8^+^NKT-like cells amplified *in vitro* was the same as the CD8^+^NKT-like cells *in vivo*.

### Enrichment of CD4 T cells, CD8 T cells and NK cells

CD4 T cells were positively selected from OT-II mouse splenocytes using the mouse CD4 Positive Selection Kit (Stemcell, BC, Canada). The CD8 T cells were positively selected from OT-I mice splenocytes using the mouse CD8a Positive Selection Kit (Stemcell, BC, Canada). panNK cells were positively selected from C57BL/6 mice splenocytes using of the mouse panNK Positive Selection Kit (Stemcell, BC, Canada). Next, the NK cells, characterized as TCRβ^−^CD49b^+^ cells, were sorted using BD FACSAria II flow cytometry.

### Confocal imaging

Different subsets were stained with CD90.2-FITC (green), LysoTracker Red (red) and Hoechst 33342 (blue). Next, images were collected as static images. However, in another experiment, the NKT_GFP_ cells were labeled with LysoTracker Red, and the GFP-DCs were used as target cells. A dynamic display of the NKT_GFP_ (red)-mediated GFP-DC (green) killing process was captured using Andor live cell confocal microscopy with a 60 ×  oil immersion lens. The yellow arrows indicate the GFP-DC morphologic changes.

### Cytokine profile analysis

CD4 T cells were enriched from OT-II mice using a mouse CD4 positive selection kit (Stemcell, BC, Canada); the cells were then sorted as CD49b^−^TCRβ^+^CD4^+^ cells. The CD8 T cells were enriched from OT-I mice using the mouse CD8a positive selection kit (Stemcell, BC, Canada); the cells were then sorted as CD49b^−^TCRβ^+^CD8^+^ cells. The CD8^+^NKT-like cells were isolated from OT-I mice using a mouse panNK positive selection kit (Stemcell, BC, Canada); the cells were then sorted as CD49b^+^TCRβ^+^CD8^+^ cells. These cells were co-cultured with DCs loaded with corresponding peptides at the ratio 10:1, and the supernatant was collected and examined using an eBioscience BMS822FF kit.

### *In vitro* antigen-specific T-cell proliferation assay

In a CD4 T cell proliferation assay, 2 × 10^6^ CD4 T cells that were magnetically isolated from OT-II mice splenocytes were co-cultured with 1 × 10^5^ GFP-DCs loaded with 200 ng/ml OVA_323–339_ peptides in 24-well plates. A total of 1 × 10^5^ NKT_GFP_ or NKT_OVA_ were added to this co-culture system, and the number of responding CD4 T cells in each group was counted after 72 hours. In a CD8 T-cell proliferation assay, 1 × 10^6^ CD8 T cells that were magnetically isolated from OT-I mice splenocytes were co-cultured with 1 × 10^5^ GFP-DCs loaded with 100 ng/ml OVA_257–264_ peptides in 24-well plates. A total of 1 × 10^5^ NKT_GFP_ or NKT_OVA_ cells were added to this co-culture system, and the number of responding CD8 T cells in each group was counted after 72 hours.

### *In vivo* adoptive transfer assay

A total of 2 × 10^6^ CD4 T cells or 1 × 10^6^ CD8 T cells were labeled with CFSE and adoptive transferred into B6 Thy1.1 mice with 1 × 10^6^ NKT_GFP_ or NKT_OVA_ cells. Next, 1 × 10^5^ GFP-DCs loaded with OVA_323–339_ or OVA_257–264_ peptides were injected. After 72 hours, splenocytes from each group of recipient mice were collected, and a CFSE dilution of CD90.2^+^CD4^+^ or CD90.2^+^CD8^+^ cells was performed.

### *In vitro* cytotoxicity assay

The OVA-DCs were stained at 4°C with CMFDA (Life Technologies, MA) at 1 μmol/10^6^ cells/mL. After a 10-min incubation, the cells were washed 3 times with PBS containing 10% FCS. A total of 1 × 10^4^ GFP-DCs and CMFDA-stained OVA-DCs were used as target cells, while the NKT_GFP_ cells were used as effector cells. The NKT_GFP_ cells were co-cultured with GFP-DCs or OVA-DCs at the E:T ratios 1:1, 5:1 and 25:1. The cells were harvested after 24 hours and incubated with 7-AAD (Life Technologies, MA) at room temperature for 10 min. The cells were then washed once with PBS and analyzed using a BD FACSAira II; 7-AAD positive cells were viewed as dead cells.

### *In vivo* immune regulation assay

A total of 2 × 10^6^ NKT_GFP_ or NKT_OVA_ cells were transferred into naïve mice, which subsequently received an immunization with 1 × 10^5^ GFP-DCs with 1 × 10^5^ OVA-DCs. Two weeks later, 5 × 10^6^ splenocytes from recipient mice in each group were isolated and stimulated by 1 × 10^4^ GFP-DCs or OVA-DCs. TNF-alpha and IFN-gamma production in each group was examined in the supernatant after 72 hours using an eBioscience BMS822FF kit.

### *In vivo* diabetes prevention assay

A total of 2 × 10^6^ NKT_GFP_ and NKT_SV_ cells were pre-injected into RIP-OVA mice, which were subsequently injected peritoneally with 8 × 10^6^ OVA-specific CD8 T cells and 2 × 10^5^ GFP-DCs loaded with OT-I peptides. The blood glucose was monitored using a One-Touch Ultra glucometer (LifeScan, CA).

### Statistical analysis

The proportion of NKT cell subsets in naïve mice versus GFP-DC-immunized mice and immune responses (shown as responder cell numbers and cytokine concentrations) in mice affected by antigen-specific CD8^+^NKT-like cells versus mice that were not given antigen-specific CD8^+^NKT-like cells were compared using a two-tailed Student’s t-test. We compared glucose concentration and body weight in NKT_GFP_-treated diabetic RIP-OVA mice versus untreated diabetic RIP-OVA mice using a Mann-Whitney U test. The replicate mice in each experiment are indicated in the figure legends. The error bars indicate the standard errors. The difference between groups was considered significant when P < 0.05.

## Additional Information

**How to cite this article**: Wang, C. *et al.* CD8^+^NKT-like cells regulate the immune response by killing antigen-bearing DCs. *Sci. Rep.* **5**, 14124; doi: 10.1038/srep14124 (2015).

## Supplementary Material

Supplementary Video S1

Supplementary Information
