# Supplementary material for: CD8+NKT-like cells regulate the immune response by killing antigen-bearing DCs
Source: Sci Rep. 2015 Sep 15;5:14124. doi: 10.1038/srep14124 (PMC4569892; doi:10.1038/srep14124)
Supplement: Supplementary Information [file srep14124-s2.doc]

**CD8^+^NKT-like cells regulate the immune response by killing antigen-bearing DCs**

Chao Wang^1,4^, Xi Liu^1,4^, Zhengyuan Li^1,4^, Yijie Chai^1^, Yunfeng Jiang^1^, Qian Wang^2^, Yewei Ji^1^, Zhongli Zhu^2^, Ying Wan^3^, Zhenglong Yuan^1^, Zhijie Chang^1^, Minghui Zhang^1^

^1^Institute of Immunology, Tsinghua University School of Medicine, Beijing 100084, China

^2^Institute of Immunology, Taishan Medical University, Taian, Shandong 271000, China

^3^Institute of Immunology, Third Military Medical University, Chongqing 400038, China

^4^These authors contributed equally to this work.

Correspondence should be addressed to M.Z. (mh-zhang@mail.tsinghua.edu.cn).

**Supplementary materials**

**
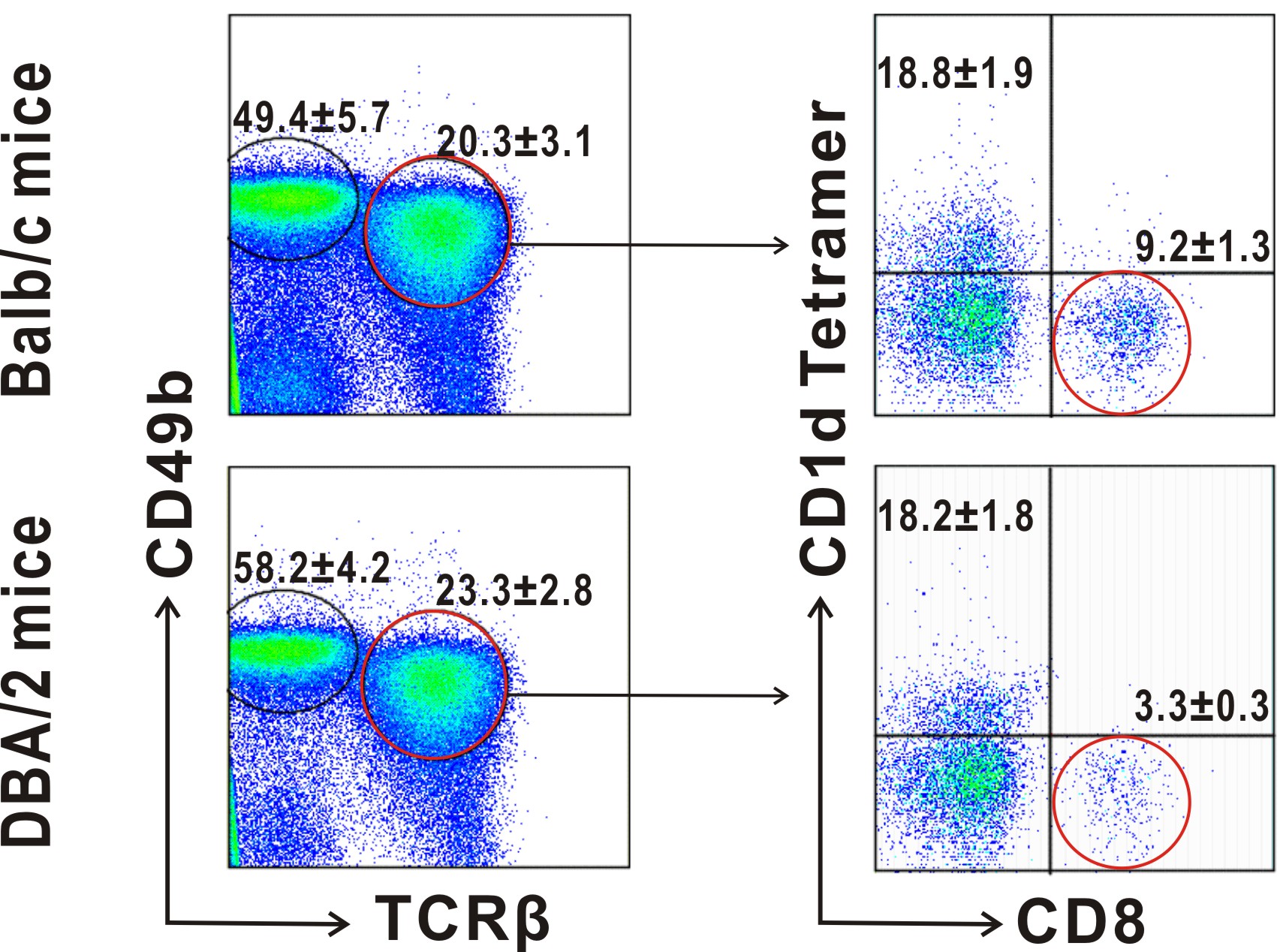
**

**
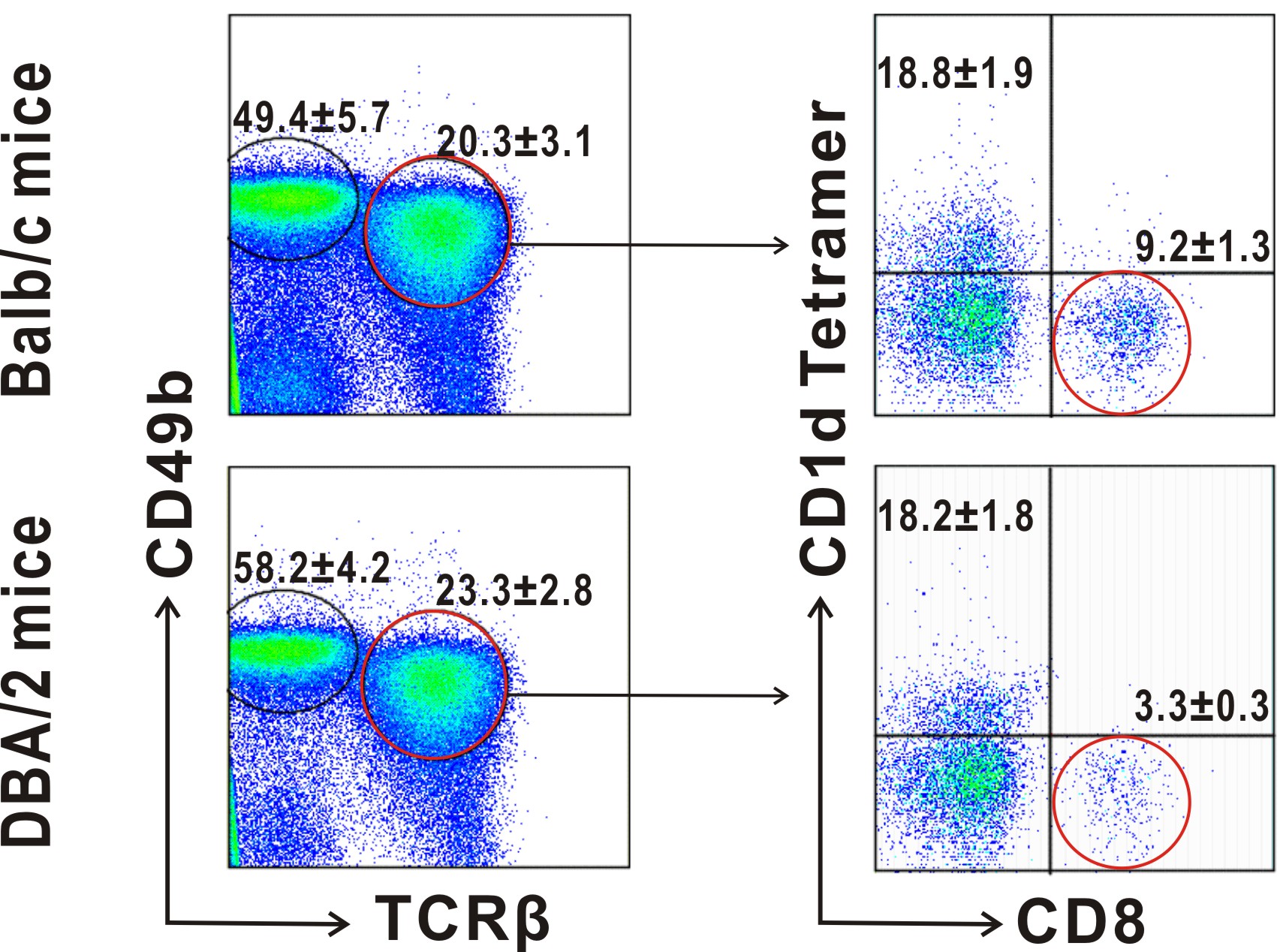
**

**Supplementary Figure S1. Examination of CD8^+^NKT-like cells in Balb/c mice and DBA/2 mice.** panNK cells were isolated from Balb/c and DBA/2 mouse splenocytes. Next, the NKT cell subsets were detected using CD8 and an α-GalCer-loaded CD1d tetramer. The data are representative of 2 independent experiments (n=8).

**
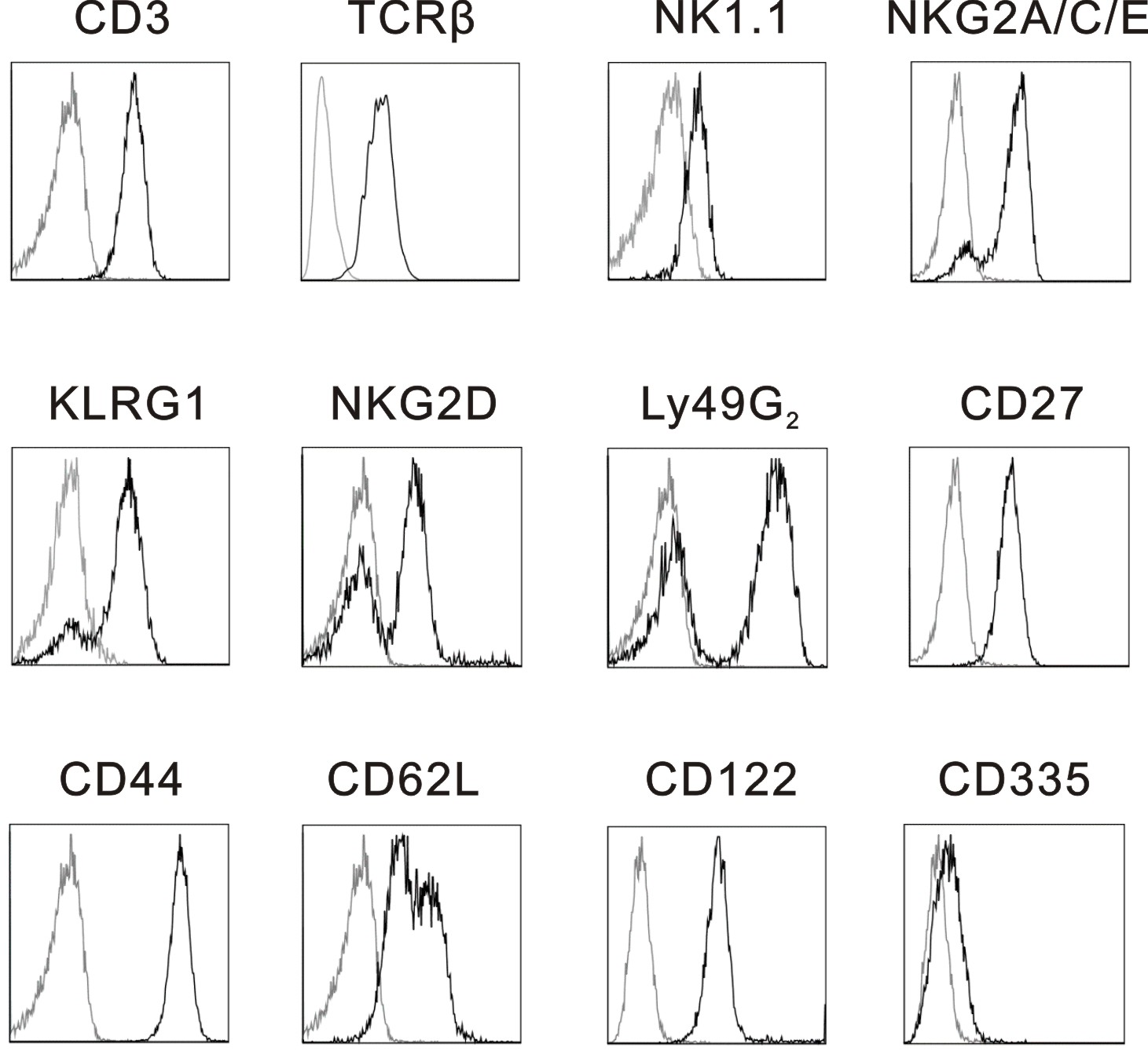
**

**Supplementary Figure S2. The phenotype of *in vitro* cultured CD8^+^NKT-like cells.** Black lines show the expression levels of the indicated markers, while the grey lines show the expression levels of the corresponding isotype controls.


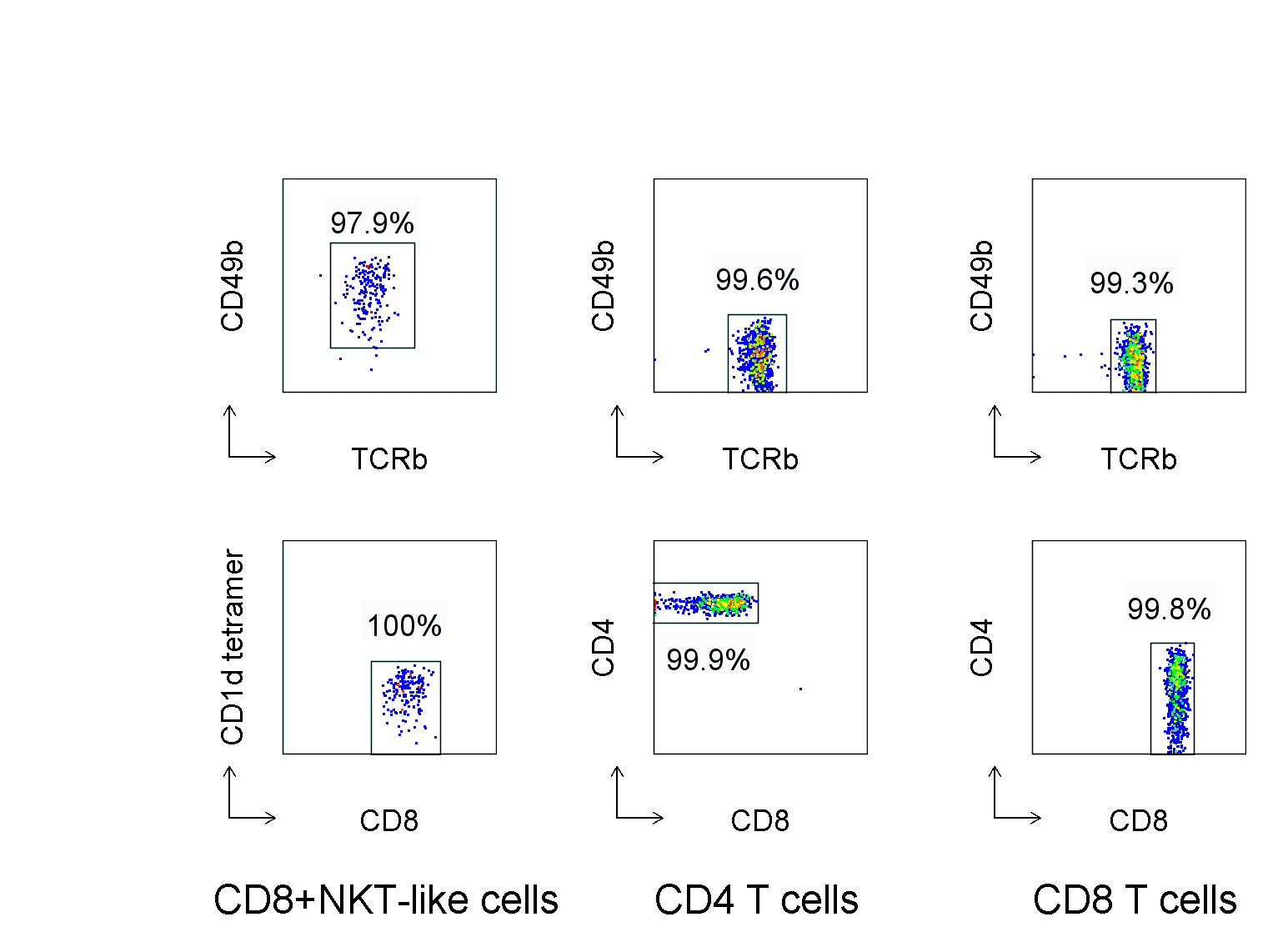


**Supplementary Figure S3. Examination of the purities of the different subsets.** CD8^+^NKT-like cells (left), CD4 T cells (middle) and CD8 T cells (right) were enriched by MACS and then sorted by flow cytometry. The purity of each subset was examined after sorting. The data are representative of 4 individual experiments and correspond to the data shown in Figure 2e.


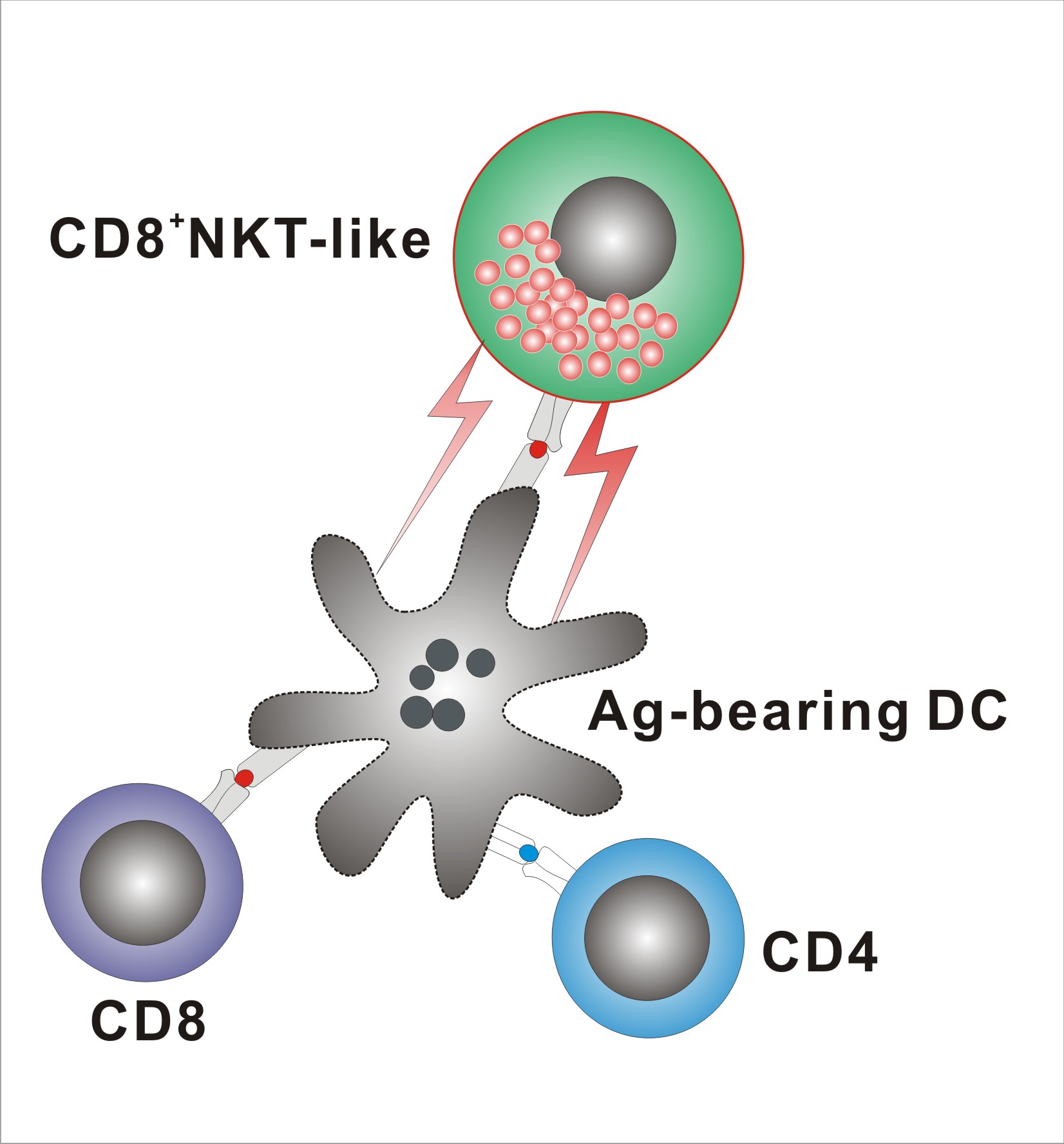


**Supplementary Figure S4. A model illustrating how CD8^+^NKT-like cells suppress the immune response.** CD8^+^NKT-like cells recognize antigens presented by DCs via their TCRs and then exhibit granule-mediated cytotoxicity against antigen-matched DCs to inhibit DC-activated CD4 and CD8 T-cell responses.


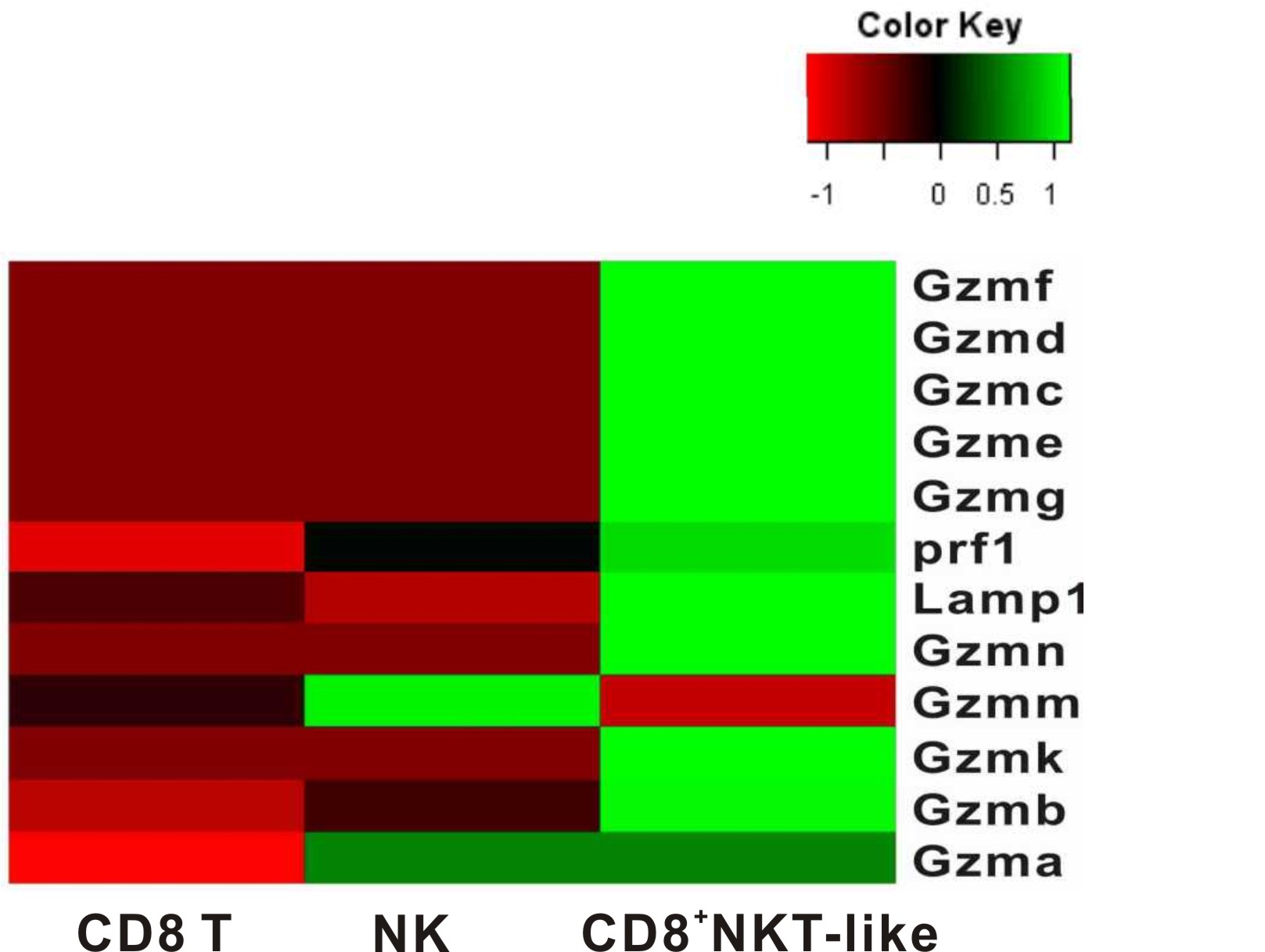


**Supplementary Figure S5. Gene chip data showing abundant granzymes in CD8^+^NKT-like cells.** Activated CD8^+^NKT-like cells, NK cells and CD8 T cells were collected for gene chips. Next, cytotoxicity-associated perforin and granzymes were compared among the cell types. The data correspond to Figure 5e.


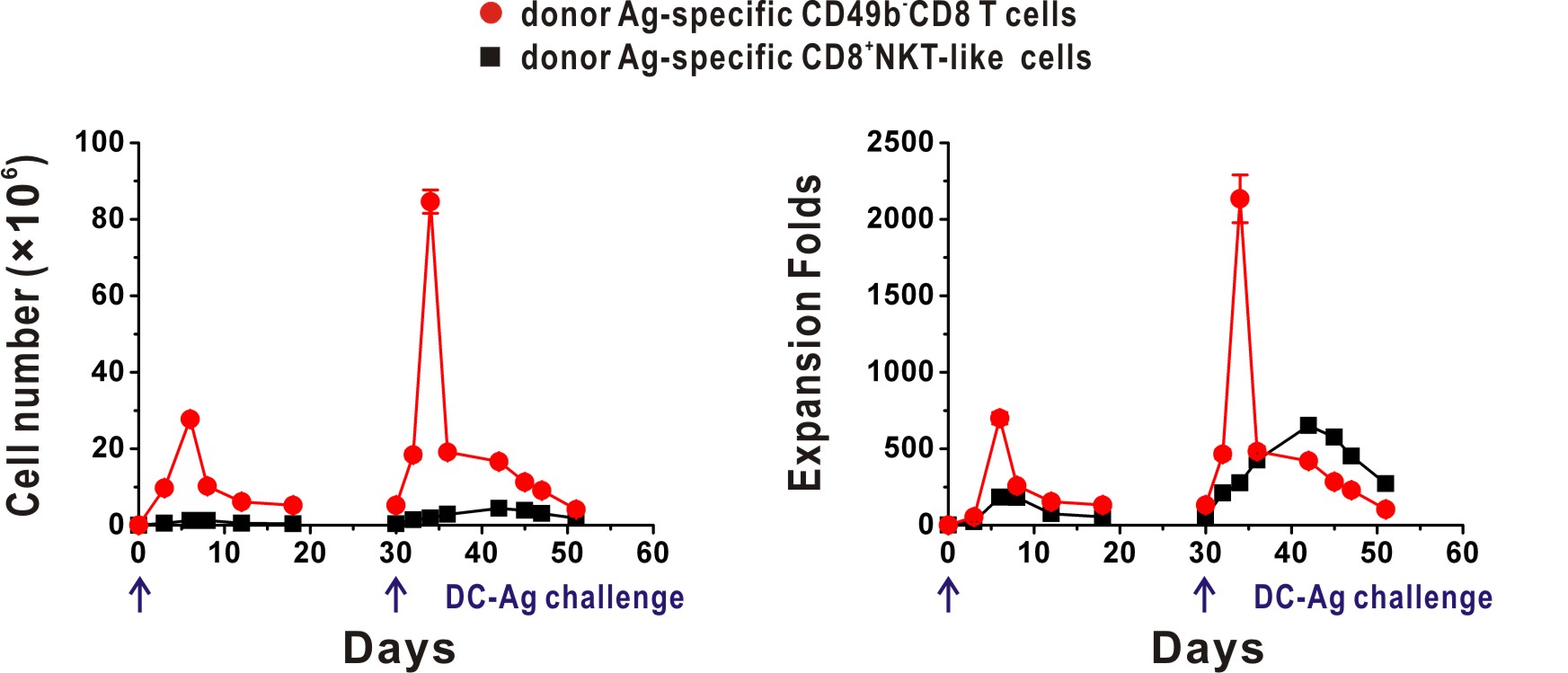


**Supplementary Figure S6. The CD8^+^NKT-like cell response lagged behind conventional CD8 T cells.** We sorted 2×10^5^ CD8^+^NKT-like cells and 2×10^6^ CD49b^-^CD8 T cells from CD45.1^+^OT-I mice, which were injected with 1×10^6^ OVA_257-264_ loaded DCs. The fold-increase and absolute cell number of the donor CD8^+^NKT-like cells and CD49b^-^CD8 T cells from peripheral blood were examined at the time points indicated. The data are representative of 3-independent experiments (n=10).


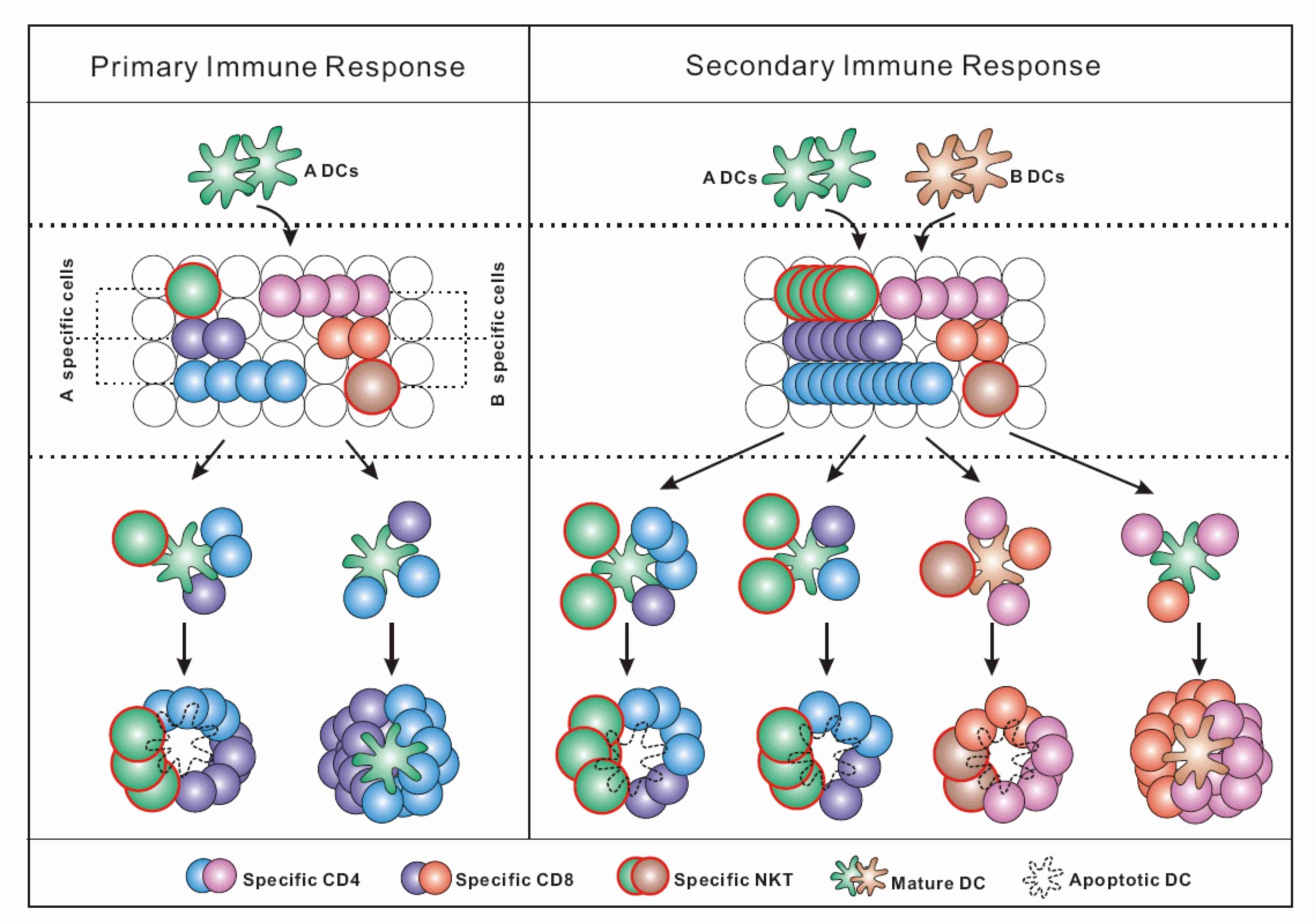


**Supplementary Figure S7. Physiological relevance of the CD8^+^NKT-like cells in the immune response.** When the cells encountered antigen A for the first time, antigen A-specific immune responses were initiated, and the number of A-specific CD8^+^NKT-like cells increased. The increased number of CD8^+^NKT-like cells more efficiently recognized and then killed A-loaded DCs. In a recall response to the same antigen A, a relatively greater number of CD8^+^NKT-like cells more rapidly recognized and killed antigen-bearing DCs, preventing an excessive immune response, which can lead to autoimmune disease.


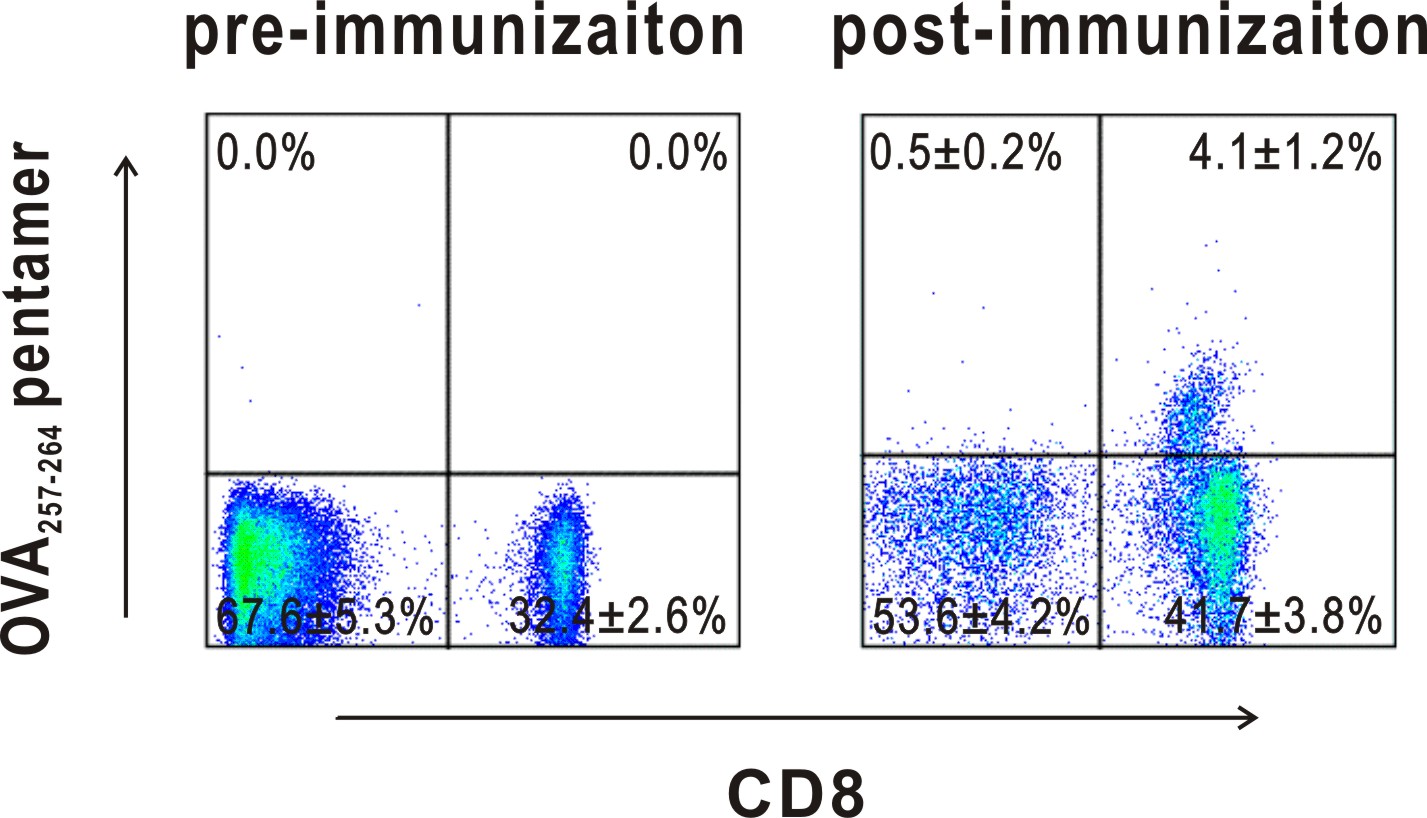


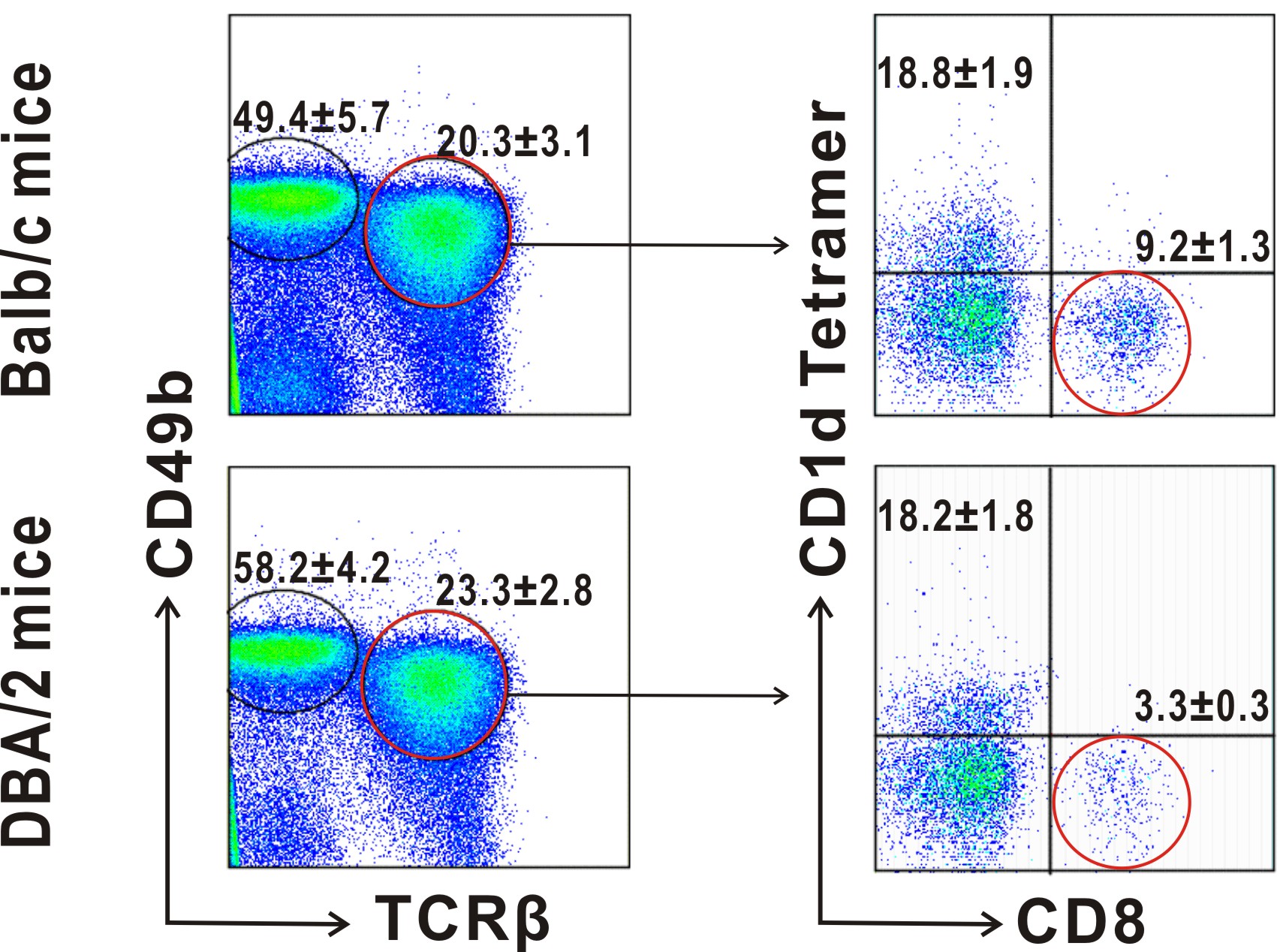


**Supplementary Figure S8. OVA-DC-immunization induced a OVA_257-264_ pentamer^+^CD49b^+^CD8 T-cell population**. C57BL/6 mice were immunized by 2×10^6^ OVA-DCs four times. Next, splenic panNK cells were isolated, and the OVA_257-264_ pentamer was labeled. The proportion in each quadrant is indicated on the plots. The data are representative of 3-independent experiments (n=8).
